# The Potentiodynamic Bottom-up Growth of the Tin Oxide Nanostructured Layer for Gas-Analytical Multisensor Array Chips

**DOI:** 10.3390/s17081908

**Published:** 2017-08-18

**Authors:** Fedor S. Fedorov, Dmitry Podgainov, Alexey Varezhnikov, Andrey Lashkov, Michail Gorshenkov, Igor Burmistrov, Martin Sommer, Victor Sysoev

**Affiliations:** 1 Laboratory of Nanomaterials, Skolkovo Institute of Science and Technology, Skolkovo Innovation Center, 3 Nobel Str., 143026 Moscow, Russia; 2 Laboratory of Sensors and Microsystems, Yuri Gagarin State Technical University of Saratov, 77 Polytechnicheskaya str., 410054 Saratov, Russia; podgaynovd@mail.ru (D.P.); alexspb88@mail.ru (A.V.); avlashkov@sstu.ru (A.L.); glas100@yandex.ru (I.B.); 3 National University of Science and Technology MISiS, 4 Leninskiy pr., 119991 Moscow, Russia; mvgorshenkov@gmail.com; 4 Institute of Microstructure Technology, Karlsruhe Institute of Technology, 1 Hermann-von-Helmholtz Platz, 76344 Eggenstein-Leopoldshafen, Germany; martin.sommer@kit.edu

**Keywords:** electrochemical deposition, gas sensor, multisensor array, tin oxide, cyclic voltammetry

## Abstract

We report a deposition of the tin oxide/hydroxide nanostructured layer by the potentiodynamic method from acidic nitrate solutions directly over the substrate, equipped with multiple strip electrodes which is employed as a gas-analytical multisensor array chip. The electrochemical synthesis is set to favor the growth of the tin oxide/hydroxide phase, while the appearance of metallic Sn is suppressed by cycling. The as-synthesized tin oxide/hydroxide layer is characterized by mesoporous morphology with grains, 250–300 nm diameter, which are further crystallized into fine SnO_2_ poly-nanocrystals following heating to 300 °C for 24 h just on the chip. The fabricated layer exhibits chemiresistive properties under exposure to organic vapors, which allows the generation of a multisensor vector signal capable of selectively distinguishing various vapors.

## 1. Introduction

Recently there is a growing interest in bottom-up growth technologies capable of synthesizing metal oxides with characteristic dimensions in the nanometer domain for gas-sensing applications [1,2]. The electrochemical deposition method fully addresses this request, yielding the oxide (nano)structures that grow directly over the metal serving as an electrode under electric bias. In terms of the method, the growth process is easy to control, and the synthesized oxide mass usually corresponds well to a consumed charge. Moreover, the electrochemical deposition allows adjusting morphology and the particular phase of the deposits, and can be performed locally following the electrode’s shape and dimensions. At the same time, the composition of the deposit can be tuned by the optimization of precursor solution composition, e.g., via adding ions to regulate the impurity level and/or changing ion concentration in the electrolyte, as well as by varying electric potential and temperature [3,4,5]. Additionally, the electrochemical deposition is a rather cost-effective method when compared to the physical methods such as molecular beam epitaxy [6], atomic layer deposition [7], chemical vapor deposition [8], etc., that is important for large-scale production protocols [9].

The NO_3_^−^ containing solutions of corresponding metal salts are frequently employed as precursors to deposit metal oxides or hydroxides in most applications. The reduction of nitrate anions under an electric potential increases a local pH value in the vicinity of the electrode due to the formation of OH^−^ ions. These processes force metal ions to precipitate in the form of hydroxides, which then might be transformed to oxides [5,10]. The method has been well adopted to mainly deposit the hydroxides and/or oxides of transition metals such as cobalt [11,12,13], nickel [14,15] and zinc [16,17,18] at a constant potential in the range between −0.75 V and −1.05 V vs. Ag/AgCl_sat_. In these systems the equilibrium potential of the metal/metal ion pair is enough negative vs. NO_3_^−^ reduction potential, or the deposition overpotentials of the metal are rather high. Moreover, the reduction of metal ions is ordinarily suppressed by the appearance of the base.

On the contrary, the electrochemical deposition of tin oxide/hydroxide is challenging because it is accompanied by the appearance of metallic Sn, whose standard equilibrium potential is −0.345 V vs. Ag/AgCl_sat_ [19]. The grown layer might have a significant Sn contribution, which should primarily depend on the [Sn^2+^]/[NO_3_^−^] ratio in the solution: the lower ratio results in the greater probability to grow tin oxide/hydroxide without a co-deposition of metallic Sn [5]. Another issue to address is a hydrolysis reaction which takes place in most of the solutions of the tin salts [20]. So, the pH value has to be adjusted to acidic one in order to suspend the hydrolysis and to yield a stable solution for the deposition which, however, restricts using low-concentrated Sn^2+^ electrolytes. Therefore, the parameters of the electrochemical deposition of this material are required to be balanced properly. To the best of the authors’ knowledge, there are only a few reports dealing with the electrochemical deposition of tin oxide/hydroxide from acidic nitrate solutions at a constant current density ranging from 1 mA/cm^2^ to 15 mA/cm^2^ [21], including a use of pulse deposition at −20 mA/cm^2^ pulses [22], or at a constant potential varying from −0.25 V to −0.6 V vs. Ag/AgCl [23,24,25]. Although the practical impact of metallic Sn co-deposition is not clearly discussed in the noted reports, the appearance of the metal might complicate an implication of the method shunting the electrodes which might be relevant, e.g., to develop gas sensors. Another approach to the tin oxide electrochemical deposition is based on the application of high power pulses to sputter the tin electrode in solution [26]. But this process is obviously energy consuming and the deposited mass might not be quantitatively controlled, since a part of the sputtered tin stays in the solution.

Here we imply a potentiodynamic method to grow simultaneously the tin and tin oxide/hydroxide nanostructured layer due to OH^−^ formation during the cathodic sweep, and to dissolve co-deposited metallic Sn at the anodic sweep. The further conversion of the deposited layer into the dioxide phase is obtained by annealing at advanced temperatures. This method is found to be suitable for growing the non-stoichiometric nanostructured tin dioxide layer over the Pt multielectroded Si/SiO_2_ substrate, which is employed as a gas-analytical multisensor array unit to selectively detect organic vapors.

## 2. Experimental

The electrochemical potentiodynamic experiments have been performed in a custom-made, three-electrode Teflon^®^ cell containing a Pt wire counter electrode and an Ag/AgCl_sat_ reference electrode (E_Ag/AgCl_ = 0.197 V vs. standard hydrogen electrode). All the potentials are quoted vs. the Ag/AgCl_sat_ reference electrode. We have employed two types of working electrodes: (1) a polycarbonate foil with sputtered Au film, ca. 0.79 cm^2^ area, which has been used to optimize the deposition parameters; and (2) Si/SiO_2_ substrate, 10 × 10 mm^2^, equipped with multiple Pt electrode strips, about 100 μm width, 4 mm length, which yield all together approx. 0.02 cm^2^ area. Also, the frontside of the Si/SiO_2_ substrate carries two Pt thermoresistors of ca. 1 μm thickness, while the backside has four meander-shaped Pt heaters of the same thickness [27,28]. These thermoresistor and heater electrodes have been free to feed by electric potential during the electrochemical growth process.

Prior to the experiments, the working electrode was rinsed with 10% HCl, cleaned with deionized water, and then dried in air. In order to carry out the CV process, the Teflon^®^ cell was filled with the solution of SnCl_2_·2H_2_O mixed with NaNO_3_, whose pH value was adjusted to 1.3 by adding 10% H_2_SO_4_ (called as a working electrolyte) to keep the electrolyte stable. The [Sn^2+^]/[NO_3_^−^] ratios were varied to be 1:2, 1:4, and 1:8, which corresponded to [Sn^2+^] concentrations equal to 0.1, 0.05, and 0.025 M, respectively, in the electrolyte. During the CV studies, we varied the potential in the range between −1.7 V and +2 V with the help of potentiostat/galvanostat (Novocontrol Alpha-AN). The CV curves were recorded at rates of 0.02, 0.05, 0.1, and 0.25 V/s, with approx. 1 mV voltage variation step. The reference experiments were performed with electrolytes containing no Sn salt at a neutral pH (equal to 7) and pH = 1.3, which are referred to hereafter as supporting electrolytes. All the chemicals used in the course of studies were of an analytical purity at least. The deposition was carried out at room temperature and repeated several times to ensure the reliability of the results.

The surface morphology and crystal structure of the grown material was characterized with scanning electron microscopy (SEM) using AURIGA CrossBeam (FIB-SEM) Workstation (Carl Zeiss) and with transmission electron microscopy (TEM), employing a JEOL JEM 1400 instrument under an accelerating voltage of 120 kV, which was equipped with a selected area electron diffraction (SAED) facility.

**Figure 1:**
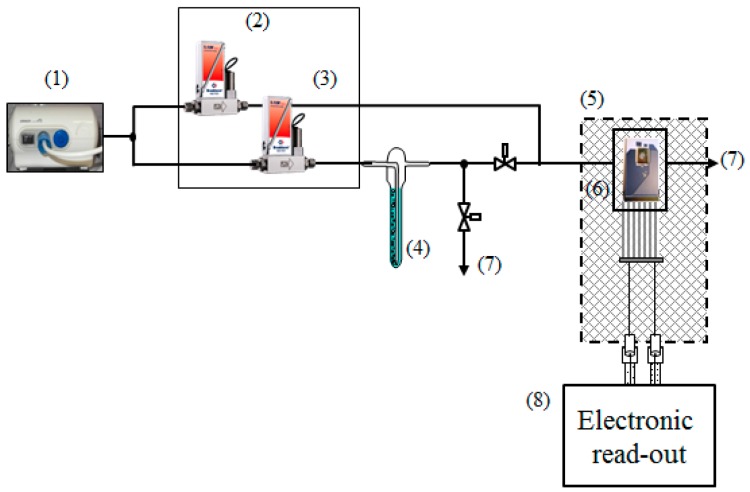
Scheme of the gas-mixing setup: (1) air compressor; (2), (3) mass flowmeters; (4) bubbler containing the analyte solution; (5) chamber; (6) multisensor array chip in steel housing; (7) gas exhaust; and (8) electrical measuring setup drawn in Figure 2 in details.

Prior to the gas-sensing measurements the tin oxide layer deposited over the multisensor array chip was stabilized at 300 °C for approx. 24 h. Then, the chip with the grown SnO_2_ layer was exposed to few organic vapors, ethanol, propanol-2, and acetone, and mixed with lab-humid (approx. 35 rel. %) air to attain 2500 ppm concentrations. Gas-mixing setup employed in the course of the studies is presented in the Figure 1. Gases to be mixed with air were generated by purging laboratory air through test liquids (acetone, ethanol, and isopropanol). We utilized an oil-free compressor to feed necessary air pressure into the system. The gas mixing unit was connected to a chamber containing the multisensor array chip. The chamber exhaust pipe released the gases to a fume hood. The air enriched with saturated test vapors in bubbler was further diluted by pure lab air to adjust the required concentration of the vapors in the mixture. The degree of dilution was controlled by mass-flowmeters. The vapor concentrations was calculated according to:

(1) $$C=\frac{P_{gas}F_{gas}}{P_{gas}F_{gas}+\left(P_{atm}-P_{gas}\right)F_{gas}+P_{atm}F_{air}}\cdot 10^{6}$$

where *P_gas_* is saturated vapor pressure, mmHg; *P_atm_* the atmospheric pressure (760 mmHg); *F_gas_* the flow rate of air via the bubbler to be enriched by analyte vapors, sccm; *F_air_* the flow rate of the pure air, sccm. Saturated vapor pressure is calculated according to:

(2) $$P_{gas}=10^{A-\frac{B}{C+T}}$$

where *A*, *B*, *С* are constants related to the employed analyte solution, and *T* the solution temperature [29].

**Figure 2:**
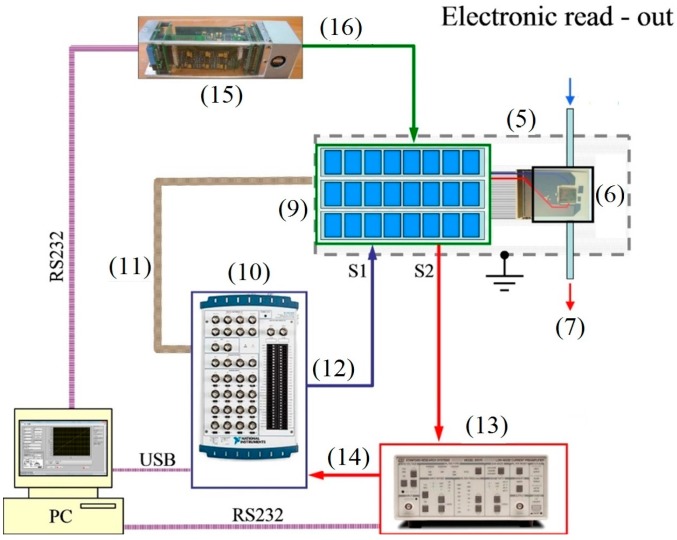
The electrical measuring setup. The part numeration follows that in Figure 1: (9) multiplexor; (10) I/O unit; (11) multiplexor control bus; (12) analog output of ±10 V; (13) current pre-amplifier; (14) analog input; (15) KAMINA unit; (16) chip temperature control line

The electrical measurements have been performed with a custom-made measuring setup which enables probing resistances up to 10 GOhm by bias from −10 V to +10 V (Figure 2). The multisensor array chip has been positioned in the sealed chamber in frames of the Faradaic cage in order to avoid the influence of external electrical fields. To sample the resistance of segments in the chip array the multiplexor has been utilized. The setup includes also I/O unit (NI-DAQ, National Instruments), which allows us to measure the input voltage and to range the output of the voltage from −10 V to +10 V with an accuracy of 0.001 V; this unit manages the multiplexor connected via the digital (TTL) output. The employed current pre-amplifier (SRS570, Stanford Research System) converts a current into output voltage at a given gain in a range from 1 mA/V to 1 pA/V. In order to control and maintain the chip temperature we have used the KAMINA unit [27], which reads out the thermoresistors values and manages the heater’s power. The whole setup is driven by personal computer (PC). We utilized USB or RS232 interfaces to connect the measuring units to the PC.

The reading of the sensor segment resistance in the chip array has been conducted by the multiplexor, which employs three units based on eight electro-mechanical relays each. The multiplexor connects coaxial outputs S1 and S2 of each pair of electrodes at the chip via the custom-made software. The multiplexor is controlled by the digital bus unit (NI-6259). During the measurements the conductance between each pair of electrodes (the sensor segments, hereafter) has been recorded in sequence with the rate down to 1 Hz for the whole array. The gases have been delivered to the chip in a continuous flow, with a rate of about 100 sccm. The chip operating temperature, up to 300 °C, has been maintained to be quasi-homogeneous over the substrate [28]. The chemiresistive response is defined as *ΔG/G_o_*, where *G_o_* and *ΔG* denote the oxide layer conductance in air and its change in the presence of organic vapors. The recorded multisensor vector signals have been processed with Linear Discriminant Analysis (LDA) [30].

## 3. Results and Discussion

### 3.1. Potentiodynamic Deposition

**Figure 3:**
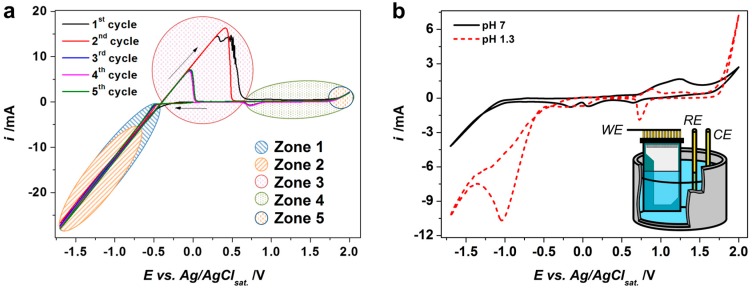
The cyclic voltammetry (CV) study of the deposition of tin oxide/hydroxide: (**a**) the CV curves recorded in working electrolyte of 0.1 M [Sn^2+^], 0.2 M [NO_3_^−^], pH = 1.3 (five cycles are presented). Zone 1: tin deposition; Zone 2: NO_3_^−^ reduction and HER: generation of base; Zone 3: tin dissolution; Zone 4: possible oxidation-reduction of Sn^2+^; Zone 5: water decomposition; (**b**) the CV curves recorded in supporting electrolyte 0.2 M NaNO_3_ at pH = 7 (black curve) and pH = 1.3 (red curve); the fifth cycles are presented; insert is the deposition scheme (WE: working electrode, RE: reference electrode, CE: counter electrode). All the curves are measured under potential scan rate of 0.05 V/s at Au working electrode, 0.79 cm^2^.

Figure 3a shows cyclic voltammetry (CV) curves obtained in the working electrolyte for the first five cycles under a scan rate equal to 0.05 V/s when employing the Au working electrode. We may distinguish several processes when the electrode potential goes in the negative direction. At about E = −0.49 V there is a substantial current change in the CV curve which seems to be associated with the deposition of metallic Sn. At the same time, the CV curves measured in a supporting electrolyte at pH = 7 indicate a steep current decrease at approx. −1 V (Figure 3b), which is shifted to about −0.54 V at pH = 1.3. This could correspond to the reduction of nitrate ions and the hydrogen evolution reaction (HER), as described elsewhere [5,31]. Both processes provoke the enhancement of the pH value in the vicinity of the working electrode surface due to generation of OH^−^ and the consumption of the H_3_O^+^ species. Such a local creation of base further yields the sedimentation of tin oxide/hydroxide.

When the potential scans back toward positive values a big anodic hump appears, starting at the value of approx. −0.47 V, which might be associated with a dissolution of metallic Sn (Figure 3a). The further enhancing of the working electrode potential toward higher positive values enforces a partial oxidation of the tin oxide/hydroxide phase, combined with an oxygen evolution reaction which corresponds to some enhancement of the current at a potential close to +2 V. The two peaks that appeared in the CV curve at E = 0.7–0.9 V at the back scan are similar to the ones observed in the supporting electrolytes (both neutral (pH = 7) and acidic (pH = 1.3) ones (Figure 3b)). So, they could be attributed to the redox processes of the nitrate intermediates. Altogether, few overlapping intervals of the potential applied to the working electrode could be identified to relate whether to a tin deposition, a tin oxide/hydroxide deposition, or the dissolution of the metallic tin as highlighted in the Figure 3a as corresponding “zones”.

**Figure 4:**
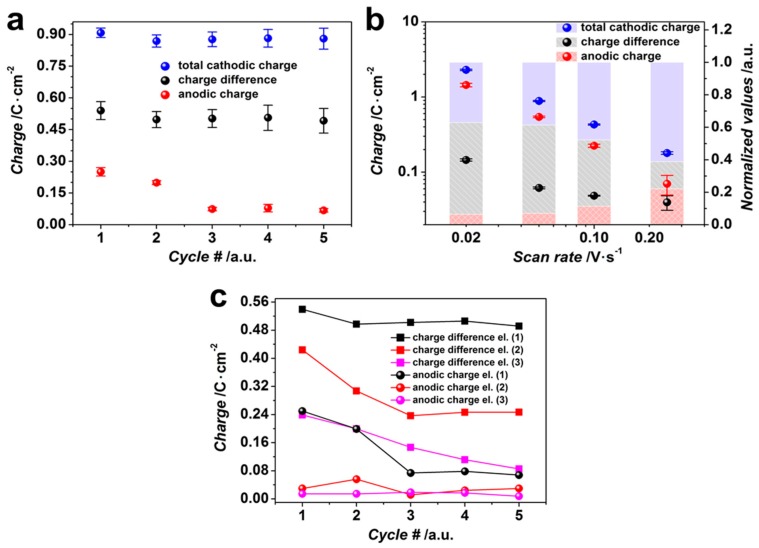
The charges calculated from CV curves: (**a**) total cathodic charge, cathodic charge difference, and anodic charge consumed in working electrolyte, 0.1 M [Sn^2+^], 0.2 M [NO_3_^−^], pH = 1.3, in dependence on CV cycle number, recorded at 0.05 V/s; (**b**) total cathodic charge, cathodic charge difference, and anodic charge consumed in working electrolyte, 0.1 M [Sn^2+^], 0.2 M [NO_3_^−^], pH = 1.3, and their values normalized to total cathodic charge in dependence on the CV scan rate; (**c**) cathodic charge difference and anodic charge consumed in working electrolytes with different [Sn^2+^]/[NO_3_^−^] ratios, 1:2 (1), 1:4 (2), 1:8 (3) in dependence on CV cycle number, recorded at the scan rate of 0.05 V/s; the lines are given as a guide for eye. (**c**) The error bars are missing for clarity reasons; the maximum deviation does not exceed 4% in case of CCD and 11% in case of anodic charge.

It is worth noting that cycling results mainly in changes of the anodic humps that appeared at the CV curves; in particular, we see a great reduction of the hump area starting from the 3rd cycle (Figure 3a). We have evaluated the charges which have been consumed under a CV cycle in the range of potentials from −0.49 V to −1.7 V (total cathodic charge) along with those which have been consumed during the anodic hump, which seem to be related to dissolution of metallic Sn. In order to extract a partial charge consumed at a single CV cycle for metallic Sn deposition, we have found the charge difference between total cathodic charge observed in the working electrolyte and the one in the supporting electrolyte (cathodic charge difference, CCD) at the same pH. The data are drawn in Figure 4a. The obtained values might hint on the impact of the Sn deposition/dissolution processes during the cycling while obtaining tin oxide/hydroxide.

As one can see, the total cathodic charge calculated for the CV curves recorded at 0.05 V/s does not change much within five cycles, showing only slight fluctuations around the value of ca. 0.89 C/cm^2^. At the same time, the anodic charge is reduced gradually over the 5 cycles with the most prominent decrease in the first three cycles from ca. 0.25 C/cm^2^ to 0.07 C/cm^2^, which remains rather stable in the following cycles. This might indicate that the growth of metallic Sn is suppressed *posteriori* the first cycle, while the oxide/hydroxide deposition is still favored. However, CCD values do not change much by cycling and fluctuate around the value equal to ca. 0.51 C/cm^2^, in line with the behavior of the total cathodic charge.

An apparent mismatch between anodic and cathodic charges might be attributed to some differences in the kinetics of the overall processes at the working and supporting electrolytes. Indeed, the nitrate reduction is usually considered to follow a series of parallel reactions which produces a number of products such as N_2_, NO_2_^−^, NH_3_, etc. [31,32], e.g.:

(3) $$NO_{3}^{-}+H_{2}O+2e^{-}\to NO_{2}^{-}+2OH^{-}\;(\mathrm{at}\;-0.187\;V)$$

(4) $$NO_{3}^{-}+3H_{2}O+5e^{-}\to \frac{1}{2}N_{2}+6OH^{-}\;(\mathrm{at}\;+0.063\;V)$$

(5) $$NO_{3}^{-}+6H_{2}O+8e^{-}\to NH_{3}+9OH^{-}\;(\mathrm{at}\;-0.317\;V)$$

The numerous stable intermediates (3–5) which appear in the multi-electronic processes occurred at the working electrode, various electrochemical and chemical reactions, including the autocatalytic ones, make the total process rather complicated [33,34]. Moreover, the mechanism of reduction of the nitrate anions is greatly influenced both by the concentration of ions in the supporting electrolyte and by the presence of alkali metal cations [35,36,37]. It is worth noting that the hydroxide phase sedimentation is usually attributed to the process (3); still, the OH^−^ species are produced in the reactions (4) and (5), which also contribute to the sedimentation of the tin oxide/hydroxide.

Along with the (3)–(5) processes, the HER reaction facilitates also, in its turn, the hydroxide sedimentation by the generation of the base because H_3_O^+^ cations are consumed and OH^−^ anions are produced [5]. Although all the processes yield tin oxide/hydroxide sedimentation, the contribution of other parallel reactions to the overall process complicates the quantitative calculation of the current efficiency related to oxide formation and can be a reason for the noted mismatch between anodic and CCD charges. In case we enhance the potential scan rate from 0.02 V/s to 0.25 V/s, the cathodic charge is reduced from ca. 2.3 C/cm^2^ to ca. 0.18 C/cm^2^, as drawn in Figure 4b. The similar trend is observed in the case of CCD and the anodic charge, which go down from ca. 1.4 C/cm^2^, 0.15 C/cm^2^ to ca. 0.07 C/cm^2^, 0.04 C/cm^2^, respectively. It is clear that while the scan rate is higher than 0.05 V/s, the CCD values are decreased and the anodic charges slightly go up compared to the total cathodic charge (see values normalized to the total cathodic charge, Figure 4b). The relative enhancing of the anodic charge when compared to the cathodic one suggests that the more prominent Sn dissolution occurs at higher scan rates, while the Sn deposition is more suppressed. Figure 4c shows the dependence of CCD and the anodic charge on the cycle number in case of electrolytes, pH = 1.3, containing different [Sn^2+^]/[NO_3_^−^] ratios, 1:2, 1:4, 1:8, where [Sn^2+^] concentrations are equal to 0.1, 0.05, and 0.025 M, respectively, at the potential scan rate of 0.05 V/s. As one can see, the lower [Sn^2+^]/[NO_3_^−^] ratios yield a lower charge consumption, which is defined by [Sn^2+^] concentration in the solution. At the same time, there is a decrease of the equilibrium potential at the Sn^0^/Sn^2+^ electrode from E = −0.383 V in the case of the 0.1 M tin (II) ion solution to E = −0.400 V in the case of the 0.025 M tin (II) ion solution, following calculations by Medusa^®^ software. It also slightly supports the suppression of the consumed charge. The anodic charges consumed in electrolytes with [Sn^2+^]/[NO_3_^−^] ratios of 1:4 and 1:8 are relatively similar; their minor magnitude indicates a negligible dissolution of metallic Sn, which corresponds to a decrease in the charge consumed due to the metal deposition. So, the lower [Sn^2+^] concentration and the higher molar fraction of [NO_3_^−^] vs. [Sn^2+^] in the electrolyte yields lower charge differences and anodic charges, i.e., it facilitates tin oxide/hydroxide deposition due to both reducing the tin (II) equilibrium potential and enhancing the current related to nitrate reduction.

We have chosen the electrolyte containing 0.1 М [Sn^2+^] and 0.2 M [NO_3_^−^], at pH = 1.3, to be cycled five times at 0.05 V/s in the range from −1.7 V to +2 V for the growth of the tin oxide layers over multielectroded substrates to serve as gas-analytical multisensor array chips. Therefore, these layers have been thoroughly characterized by electron microscopy.

### 3.2. Layer Morphology and Crystal Structure

**Figure 5:**
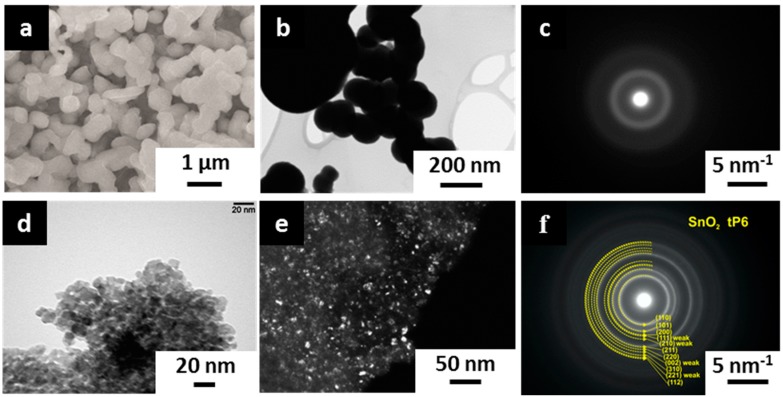
The electron microscopy characterization of the tin oxide/hydroxide layer deposited by potentiodynamic method in working electrolyte, 0.1 M [Sn^2+^], 0.2 M [NO_3_^−^], pH = 1.3, under potential scan rate of 0.05 V/s, five cycles: (**a**–**c**) as-grown state; (**d**–**f**) following heating at 300 °C for 24 h in lab air conditions. (**a**) SEM image, (**b**–**e**) bright field and dark field TEM images, (**c**,**f**) SAED images.

The SEM inspection reveals that the tin oxide/hydroxide layer, as-deposited under the chosen parameters, possesses a highly porous mesoscopic morphology with grains of 250–500 nm size (Figure 5a). Some of the spherical grains tend to be agglomerated. It is worth noting that there is no sign of the metallic Sn crystals appearance in the obtained deposit. The spherical shape of the grains is further verified by TEM images taken in a bright field mode (Figure 5b), which also indicates the non-existence of well-shaped crystals. The SAED diagram further proves an amorphous structure of the as-deposited tin oxide/hydroxide layer (Figure 5c). Following the heating up to 300 °C for 24 h in lab air conditions, this layer is crystallized into the polycrystalline tin dioxide layer with fine particles up to 25 nm in size (Figure 5d,e). When compared to as-deposited layer, the SAED pattern of the heat-treated layer gives much stronger clear-shaped lines which correspond to d-values of 3.354 Å, 2.641 Å, 1.758 Å, and 1.425 Å at (110), (101), (211), and (112) planes of tin (IV) oxide (Figure 5f). The weak line, which corresponds to d = 2.299 Å, is due to a diffraction at the (111) plane. Still, the small crystallite size yields a broadening of the rings in the SAED pattern.

Thus, the obtained crystalline structure of the deposited SnO_x_ layer is in the geometry domain, where the surface chemisorption processes could significantly influence the free carrier concentration [38,39] which appeared primarily due to intrinsic defects [40] that favors its use as a gas-sensitive one.

### 3.3. Sensor Performance

**Figure 6:**
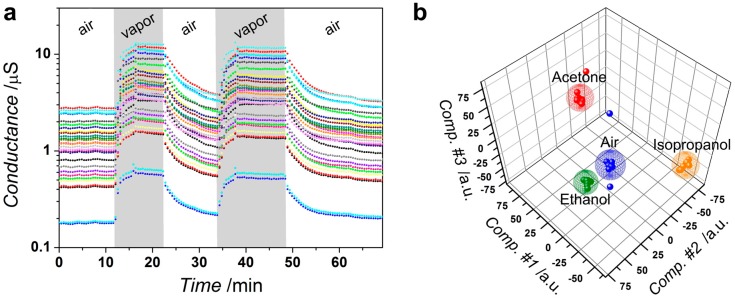
The gas-sensitive properties of the SnO_x_ layer grown over multielectrode chip by potentiodynamic method in working electrolyte, 0.1 M [Sn^2+^], 0.2 M [NO_3_^−^], pH = 1.3, under potential scan rate of 0.05 V/s, five cycles. The operating temperature is 250 °C: (**a**) the conductance transient of sensor segments under exposure to acetone: “air”, “vapor” denote pure lab humid, approx. 35 rel. %, air and mixture of acetone, 2500 ppm concentration, with the air, respectively; (**b**) Linear Discriminant Analysis (LDA) processing of the vector signals generated by the sensor segment array toward the pure air and organic vapors, ethanol, propanol-2, acetone, of 2500 ppm concentration, mixed with the air.

We have studied the local conductance of the deposited tin oxide layer located between each pair of multiple Pt electrodes, ca. 80 μm gap, as a part of the multisensor array chip. The operating temperature has been installed to be equal to 250 °C, which has been found to be an optimal one to observe the chemiresistive effect in tin dioxide [41]. Here we show, as an example, the acetone-induced transient of the conductance of the deposited SnO_x_ layer segments in the chip (Figure 6a). As one can see, the conductance is increased in the presence of the organic vapors and restores to initial values in air. This agrees well with the known *n*-type behavior of non-stoichiometric SnO_2_ when the chemisorption of reducing agents reduces oxygen coverage of the tin oxide surface and the corresponding at-surface space charge region [42]. The similar results have been observed in the case of the 2-propanol and ethanol vapors. The segments of the deposited SnO_x_ layer have quite a high chemiresistive response, 225–400%, vs. to 2500 ppm of organic vapors. The response variation is primarily caused by differences in the percolation paths through the polycrystalline networks in the inter-electrode gaps. The varied interplay of magnitude of the potential barriers at the SnO_x_ crystal interfaces and the depletion of the crystals at each chip segment yields a further contribution to the segment characteristic response [43]. That allows one making of the multidimensional vector response of the chip when combining the signals from each segment in the array. In contrast to the response of the single segment at the chip, which is fundamentally unselective to reducing gases as a feature of wide-band oxide [44], the multidimensional response could be selective [45]. With that purpose, the chip vector signal is processed with the pattern recognition technique in order to selectively identify the kind of the test vapors (see, for instance, reference [46]).

Here, we employed LDA to process the signal from the deposited SnO_x_-based chip. Prior to feeding the LDA algorithm each segment conductance has been normalized by median value, calculated from the segment array located at the chip. The LDA has been carried out to build clusters of the data framed by spheres [29]. Figure 6b shows the calculated 3-component LDA space, where the chip vector response to the three test organic vapors of the same (2500 ppm) concentration is projected to. As one can see, the data points tend to group into clusters corresponding to each of the test vapors, which do not intersect and are well separated; the median Mahalanobis distance between cluster centers is approx. 98.2. That means that these multidimensional data could be employed for selective identification of the vapors, even the chemically akin (as ethanol and 2-propanol) ones.

## 4. Conclusions

Thus, we show here the possibility to deposit tin oxide/hydroxide nanostructured layers by the potentiodynamic method from nitrate solutions, which facilitates the growth of tin and tin oxide/hydroxide due to the generation of the base under the cathodic sweep and the dissolution of metallic Sn afterwards at the anodic sweep. The process is forced by cycling at rather high scan rates, up from 0.05 V/s. It depends on the Sn^2+^-to-NO_3_^−^ molar ratio with the optimum at 0.1 M Sn^2+^ to 0.2 M [NO_3_^−^], when the potential is cycled in the range of −1.7 V to +2.0 V vs. Ag/AgCl_sat_ with a scan rate of 0.05 V/s. The tin oxide/hydroxide growth is favored by the increase of the cycle number. The as-grown tin oxide/hydroxide layer is present in an amorphous phase, which possesses mesoscopic morphology with characteristic grains of 250–500 nm size. By stabilizing the layer grown over the chip at 300 °C for 24 h, the material is transformed into nanocrystalline oxygen-deficient tin (IV) oxide, which is found to be sensitive to organic vapors at the ppm concentration range. The selectivity of the vapor identification is approached by processing the vector signal from an array of sensor segments in the chip by the pattern recognition technique, according to the multisensor concept.
